# Intravascular Lymphoma Associated with the Female Genital Tract—Diagnostic Considerations, Therapeutic Approaches, and Outcomes

**DOI:** 10.3390/diseases14030109

**Published:** 2026-03-17

**Authors:** Aleksandar Ristic, Marija Rovcanin, Ana Tomic, Aleksandar Rakic, Nebojsa Zecevic, Svetlana Jankovic

**Affiliations:** 1 Clinic for Gynecology and Obstetrics, Narodni Front, Kraljice Natalije 62, 11000 Belgrade, Serbia; 2 Faculty of Medicine, University of Belgrade, Dr Subotica Starijeg 8, 11000 Belgrade, Serbia; 3 Center for Radiology, University Clinical Center of Serbia, Pasterova 2, 11000 Belgrade, Serbia

**Keywords:** intravascular lymphoma, female, genital tract, uterus, ovary

## Abstract

Intravascular lymphoma (IVL) is an uncommon subtype of non-Hodgkin’s extranodal lymphoma, distinguished by the proliferation of neoplastic cells within the lumen of small- to medium-sized arteries, with various organs recorded as impacted. The objective of this study was to evaluate the current literature about IVL and its involvement in the female genital tract, including an overview of diagnostic methods, imaging, and pathological features, selected therapy modalities, and outcomes in patients afflicted by this malignancy. We performed a narrative review with a systematic identification and presentation of published cases of IVL affecting the female genital tract. A literature search was carried out across PubMed, Scopus, and Web of Science for relevant studies presenting data on IVL affecting the female genital tract. Case reports and series that met predefined inclusion and exclusion criteria specified by the modified PECOS (“Population,” “Exposure,” “Comparison,” “Outcomes,” and “Study design”) framework were included. Patients most commonly presented with abnormal vaginal bleeding, pelvic pain, and B symptoms. Fluorodeoxyglucose positron emission tomography computed tomography (FDG PET/CT), often performed alongside abnormal laboratory findings such as elevated lactate dehydrogenase (LDH), played a key role in raising suspicion for hematologic involvement of the female genital tract and guiding biopsy. Most cases represented B-cell intravascular lymphoma and were treated with Rituximab plus (CHOPR-CHOP) based chemotherapy, frequently combined with hysterectomy.

## 1. Introduction

Intravascular lymphoma (IVL) is an uncommon and aggressive variant of non-Hodgkin’s lymphoma (NHL), distinguished by the proliferation of neoplastic cells confined to the lumen of small- to medium-sized arteries [1]. Various subtypes are identified based on the predominant clinical manifestation. The classic subtype, more prevalent in Western countries, is characterized by B symptoms (unexplained fever, night sweats, and unintentional weight loss) along with localized symptoms associated with the primarily affected organ. The cutaneous subtype is limited to the skin at presentation and is characterized as less aggressive. The hemophagocytic subtype is more commonly observed in Asian individuals, with symptoms associated with hemophagocytic syndrome prevailing, resulting in a poorer prognosis [2].

Any organ may be implicated, but the central nervous system (CNS) [3,4] and skin [1] are reported as the two most frequently afflicted areas. These patients have neurological impairments and cutaneous manifestations, including petechiae, purpura, plaques, and discoloration. Other often documented affected organs encompass the adrenal glands [5], pituitary gland [6,7], lungs [8], spleen and liver [9], and kidneys [10]. However, the female genital tract is rarely impacted. Interestingly, bone marrow, blood, and lymph nodes are considered to be typically spared.

IVL is considered an uncommon condition, with 0.095 cases per million. Nonetheless, the incidence of IVL saw a marked increase at the onset of the 21st century [11]. Most reported IVLs are of B-cell lineage and are defined as a subtype of non-Hodgkin’s extranodal diffuse large B-cell lymphoma, as per World Health Organization (WHO) classification [12]. Rarely reported types are T-cell lymphoma, Hodgkin lymphoma [13], and NK (natural killer)/T-cell lymphoma [14,15].

This malignancy is inadequately comprehended and has consistently been recognized as an aggressive disease with adverse outcomes. Consequently, a portion of documented patients receive a diagnosis post-mortem [16], within a year of disease onset [17]. Current clinical practice suggests that completely dependable staging guidelines for IVL are lacking. Nonetheless, as our understanding of this perplexing disease expands, the diagnostic rate among living patients is improving. Furthermore, advancements in diagnostic techniques, including immunohistochemical biomarkers and innovative imaging methods that evaluate metabolic activity, such as fluorodeoxyglucose positron emission tomography computed tomography (FDG PET/CT), have facilitated the timely identification of this lymphoma subtype in clinical practice [18].

The objective of this review was to evaluate the existing literature on IVL and its involvement in the female genital tract, including a summary of documented cases, diagnostic methods employed, imaging and pathological characteristics, selected therapeutic strategies, and patient outcomes associated with this malignancy.

## 2. Materials and Methods

### 2.1. Search Strategy and Data Screening

This article is a narrative review with a systematic identification and presentation of published cases of IVL affecting the female genital tract.

A comprehensive electronic literature search was performed across Medline (via PubMed), Scopus, and Web of Science for relevant studies presenting data on IVL affecting the female genital tract. A combination of the following medical subject titles, keywords, and targeted phrases was utilized: “intravascular lymphoma,” “female,” “uterus,” “uterine,” “ovary,” “fallopian tube,” “vagina,” “adnexa.” A filter was applied to search for available full-text English publications. Selected article types were reported cases and case series in the human population, with no time limitations. The final search was performed on 15 December 2025. Titles and abstracts were initially evaluated, and possibly pertinent papers were selected and assessed for eligibility criteria. A PRISMA (Preferred Reporting Items for Systematic Reviews and Meta-Analyses) flow diagram was not applied due to the exclusive inclusion of case reports and small case series, which precluded formal quantitative synthesis.

### 2.2. Eligibility

Studies that met predefined inclusion and exclusion criteria specified by the modified PECOS (“Population,” “Exposure,” “Comparison,” “Outcomes,” and “Study design”) framework were deemed eligible for the data extraction and further analysis. The population included female patients of any age diagnosed with IVL (P). The exposure of interest was IVL of the female genital tract (E). No comparator was required due to the descriptive nature of the included studies (C). Outcomes included clinical features, laboratory findings, diagnostic evaluations, treatment of living patients, and outcomes (O). Eligible study designs were case reports and case series (S).

### 2.3. Data Extraction and Synthesis

Data extracted included the author(s), the year of publication, and country where the case was reported. Case data included patients’ characteristics and disease-related information. Specifically, information concerning patient age, tumor location (uterus/ovary/vagina), IVL subtype (B-cell, T-cell, or NK/T-cell), clinical presentation, laboratory findings, diagnostic methods (histopathological investigation and/or imaging), treatment provided, and outcomes were recorded. Descriptive analysis of noted findings was implemented. Owing to the variability of the presented data, a narrative synthesis approach was utilized. We employed a thematic grouping strategy by clinical and laboratory findings, diagnostic approach, and treatment strategies with outcomes in order to facilitate meaningful narrative synthesis despite the heterogeneity in reported features and outcomes. Moreover, we reported the number of cases and calculated frequencies detected with the chosen findings. Comparative tables were constructed to demonstrate variations between cases and clinical features and outcomes.

## 3. Results

### 3.1. Study Characteristics

A total of 16 publications were included in the in-depth analysis, and they are presented in Table 1 in chronological order. Fifteen studies presented with single case reports, while only one study [19] presented a case series of 5 patients, compiling a total of 20 patients with IVL involving the female genital tract.

### 3.2. Clinical and Laboratory Features

Clinical and laboratory features of reported patients from Table 2 are presented in Supplementary Table S1.

The mean age of all reported cases was 62.3 years, with the youngest case at 43 years and the oldest at 75 years.

Out of all B symptoms, fever was the most reported symptom of noted studies—more precisely in 14 (70%) cases [19,22,23,24,25,27,29,32,33,34]—followed by rapid weight loss in 7 (35%) cases [21,22,26,27,29,30,31] and night sweats in 3 (15%) cases [22,29,30], joined by malaise and weakness in 3 (15%) cases [21,23,24]. Only one study reported an asymptomatic patient with proven IVL in the uterus and ovaries [28]. Gynecological symptoms suggestive of genital tract involvement, including irregular uterine or vaginal bleeding, were documented in five studies, in exactly five (25%) cases [20,21,25,27,31], along with chronic pelvic pain in one (5%) case [32].

Anemia and thrombocytopenia were reported in three studies as the main blood count disorders, with anemia reported in 12 (60%) cases [19,21,22,25,29,32,33,34] and thrombocytopenia in 6 (30%) cases [19,25,31,34]. Among all biochemical indicators, Lactate Dehydrogenase (LDH) was elevated in 13 (65%) cases [19,23,25,26,28,29,30,31,32,33], with two studies reporting elevated C-reactive protein (CRP) in 7 (35%) cases [19,21,22,27,29], as well as an increase in soluble form of the soluble interleukin-2 receptor (sIL2) in 3 (15%) cases [21,23,25].

### 3.3. Imaging Features, Gynecological Examination, and Diagnostic Procedures

Only one study reported the use of vaginal ultrasound, showing a thickened endometrium with scattered irregular echoic areas [27]. Computed tomography (CT) was utilized to rule out enlarged lymph nodes, spleen, or liver in three studies [20,23,25], with one case documenting a heterogeneously enlarged uterine cavity [22] and a leiomyomatous lesion in the uterus [23].

Magnetic Resonance Imaging (MRI) of the pelvis was conducted in a number of studies. However, a small number of studies revealed significant findings indicative of uterine malignant pathology, characterized by abnormal enhancement in the left-sided middle myometrium of the uterus [34]. Ong et al. [33] reported one IVL case that presented with an 11 cm uterine mass with lymphomatous obstruction.

FDG PET/CT was used in both diagnostic evaluation [19,22,26,28,34] and treatment efficacy assessment through detection of residual post-treatment FDG avidity [30,31,34]. Reported diagnostic findings presented as focally increased FDG uterine uptake in 10 (50%) cases [19,22,26,28,30,34] or less commonly widespread uptake in the uterus, cervix, and vagina [26], accompanied by hypermetabolic activity in the lymph nodes [19,26].

With the aforementioned gynecological symptoms, as well as abnormal FDG uptake, patients reportedly underwent different gynecological examinations (Table 3).

In reported cases, diagnostic approaches were heterogeneous and included uterine curettage [20,23,30], endometrial cytology [23,25], and endometrial biopsy [19,23,29]. Vaginal mucosal biopsy was performed in one case [26], while vulvar mass biopsy was used to prove the only noted case of vulva-affected IVL [24].

Uterine curettage yielded various outcomes, identifying an undifferentiated malignant tumor [20], establishing a conclusive diagnosis of large cell lymphoma [22,30], but in some instances, it failed to detect malignant cells [23]. Additionally, other studies reported the detection of malignant (hematopoietic and/or non-epithelial) cells in certain cases by performing endometrial cytology [23,25,28] and endometrial biopsy [19,23,29]. However, the overall diagnostic value was variable, ultimately requiring hysterectomy to establish the diagnosis [19,20,28,30].

From a gynecologic oncology perspective, intravascular lymphoma involving the female genital tract represents a major diagnostic challenge, as it may clinically and radiologically mimic primary gynecologic malignancies, including endometrial carcinoma, uterine sarcomas, and metastatic disease. The absence of a discrete tumor mass, lack of significant lymphadenopathy, and nonspecific imaging findings often delay diagnosis. Therefore, intravascular lymphoma should be considered in elderly patients presenting with unexplained gynecologic symptoms accompanied by systemic B symptoms and abnormal laboratory parameters, particularly elevated LDH, even in the absence of overt malignant features on conventional imaging.

### 3.4. Histopathology and Immunophenotype

In studies reporting immunophenotype (a total of 10), as shown in Table 4, all observed patients were CD20+, and eight were CD79a+. CD5 exhibited positivity in seven instances. CD10 yielded negative results in six instances. Three studies did not test for CD79a+, CD5, and CD10, respectively. BCL-2 exhibited positivity in four instances, with a singular reported case demonstrating positivity for both BCL-2 and MYC. Ki-67 was reported as high in five cases, and all documented Ki-67 indices were elevated at a minimum of 80% where specified [20,26,27,32].

Among all documented studies, normal bone marrow was documented in six studies [19,20,21,23,25,26]. Hypoplastic marrow, but with no evidence of IVL [27] or with mild dysplastic changes [31], was reported in a small portion of patients. Hyperplastic bone marrow with lymphoid aggregate was registered in one study [30], whereas hemophagocytic lymphohistiocytosis was identified in three cases [30,31,34].

### 3.5. Treatment and Outcomes

The main reported chemotherapy protocols and treatment modalities are presented in Table 5. All patients received chemotherapy, mainly receiving a standard cyclophosphamide, doxorubicin, vincristine, and prednisone with rituximab (R-CHOP) protocol [20,21,22,23,25,26], with surgery (total hysterectomy (THO) with or without bilateral salpingo-ovariectomy (BSO)) performed in the majority of reported cases [20,21,25,26,28,30,33,34]. Supplementary Table S2 shows further documented chemotherapy (preventive intrathecal methotrexate, etoposide, filgrastim, etc.), as well as additionally performed procedures such as autologous stem cell transplantation.

A favorable outcome, defined as clinically and/or laboratory-confirmed disease remission (RM), was achieved in 14 (70%) reported cases. Death by the disease was registered in three cases [19,22,29]. One patient reportedly developed neutropenic sepsis and died 2 weeks after diagnosis [26], while one patient developed another primary (endometrial) carcinoma and died 8 months after the initial IVL diagnosis [31].

## 4. Discussion

IVL of the female genital tract is a true rarity, hereby presented in small cohort of 20 patients across the current literature. Few studies have examined the intrinsic characteristic of IVL, the dynamics of its abnormal proliferative growth within blood vessel lumina, and its possible causes. Nevertheless, as we presently understand, IVL cells lack superficial markers which are necessary for extravasation of lymphocytes and therefore impair transmigration across the endothelium [35]. Any involved organ typically pathologically presents with ischemia and hemorrhagic or necrotic lesions as a result of vascular blockage brought on by further IVL cell proliferation [36]. The literature detailing the pathogenesis of female genital tract IVL is exceedingly limited and primarily comprises case reports or short clinicopathologic series. Consequently, identified mechanisms such as vascular blockage, hemorrhage, and ischemia are the most likely causes of uterine artery weakness, potentially resulting in irregular bleeding. Additionally, IVL cells express molecules that allow them to adhere to the endothelium but lack those involved in extravasation, which prevents them from forming extravascular masses [37] that are visible on traditional imaging modalities like US, CT, or MRI [35]. However, their pathological metabolic activity means that FDG PET/CT is of great assistance in various IVL cases [38].

The median age at diagnosis of IVL is most often in the seventh decade, as previously documented [11,39]. Two predominant clinical clusters are evident in patients with female genital IVL in the cases presented. The primary and most common manifestation consists of isolated B symptoms, occurring in 70% of reported cases [19,22,23,24,25,27,29,32,33,34], with further clinical course detection of anemia [19,21,22,25,29,32,33,34] and elevated LDH [19,23,25,26,28,29,30,31,32,33] in over 60% of reported cases, consistent with the systemic biological behavior typically observed in lymphoproliferative disorders, as well as IVL cases in particular [40]. With the affection of the female genital tract, predominately the uterus, systemic symptoms in presented cases tend to be associated with gynecological signs, particularly irregular uterine hemorrhage [20,21,25,27,31]. Despite this group constituting a smaller fraction of the reported symptomatology, this clinical cluster should be acknowledged as a significant marker for additional gynecological assessment, particularly in postmenopausal women, warranting further diagnostic investigation, including gynecological evaluation and FDG-PET/CT, to facilitate prompt diagnosis and the commencement of suitable treatment.

As previously mentioned, anemia was the most common abnormal laboratory finding in IVL cases [39,41], usually occurring without leukocytosis [41], followed by thrombocytopenia [39,41]. Elevated concentrations of LDH, serum sIL-2R, and CRP have frequently been recorded in prior studies that explored cases with IVL [41,42]. Increased LDH is recognized as a crucial marker for lymphoma activity and tumor burden [43].

Serum sIL-2R is recognized as a highly effective supplementary diagnostic marker for lymphoma patients, especially those with fever. The tumor volume in T-cell lymphoma is indicated by the proteolytic dissolution of the membrane-bound interleukin-2 receptor α-chain on activated T-cells; thus, its elevation signifies T-cell activation and immunological dysregulation [44]. However, T-cell lymphoma is a true rarity when it comes to IVL of the female genital tract. Therefore, an increase in sIL-2R in B-cell type IVLs is becoming acknowledged for its diagnostic and prognostic significance, as the heightened serum levels of sIL-2R and LDH, along with the prevalence of B symptoms, seemingly indicate the tumor growth milieu of IVL in the peripheral blood [45].

The nonspecific and diverse symptoms, in the general absence of significant lymphadenopathy, as well as visible and detectable skin lesions, can result in critical delays in diagnosing this clinically aggressive lymphoma. It is a recognized concept that B-cell-type IVL typically cannot be diagnosed with CT or MRI due to the absence of associated solid lesions, as only one paper showed reported cases that presented with an MRI uterine mass [33]. CT has rarely been reported to detect important details that may point to uterine pathology, with only one detected case of uterine cavity enlargement [22]; however, it is utilized for excluding hepatosplenomegaly and lymphadenopathy. This is in accordance with previously shown, rather low detection rates for abnormal findings in uterine cancer, as it has more value for detecting extra-uterine manifestations of the disease [46]. Moreover, as previous cases of other site IVLs have reported normal liver and spleen [47] with the absence of lymphadenopathy [47,48], these findings should not immediately reject the diagnosis.

In the described cases, diagnostic methods were varied and encompassed uterine curettage, endometrial cytology, and endometrial biopsy, with the detection of malignant hematopoietic cells in certain reports and inconsistent overall diagnostic yield, with some cases ultimately requiring diagnostic hysterectomy. There are conflicting reports regarding a conclusive diagnosis method for gynecological hematopoietic cancers [49,50,51,52]. These malignancies should be suspected if there are unexplained gynecological and systemic B symptoms, particularly in the setting of abnormal blood tests, notably anemia and increased LDH, whether or not there is a detectable uterine and/or ovarian mass or lymphadenopathy. Documented clinical and biochemical indicators should be meticulously examined, as they may mimic other gynecological malignancies [53], principally necessitating advanced investigations such as FDG PET/CT.

FDG PET/CT has been deemed the most clinically significant modality for assessing patients presenting with IVL, revealing hypermetabolic activity in the pelvic region [19,22,26,28,34]. Accordingly, studies have previously reported hypermetabolic sites of primary IVL involvement in the liver [54], spleen [41,54], regions of skin lesions [41], lungs [54,55], kidneys [54,55], and bones [55]. Consequently, in the context of suspected hematopoietic malignancy—specifically IVL in this case—18F-FDG PET/CT scanning is demonstrated to be exceptionally useful, valuable, and sensitive for diagnosis [42]. Despite the fact that FDG PET/CT scans are frequently utilized for gynecological hematopoietic cancer staging and treatment evaluation [52], in IVL instances, the outcomes of the FDG-PET scan ultimately direct the physician in the direction of the best biopsy site, despite the SUVmax values in IVL cases being less pronounced than in other lymphoma variants [56].

IVLs are majorly reported as a B-cell lineage with CD20+ markers [57], commonly also with CD79a+ [58,59], but rarely with PAX5+ [60]. Moreover, the vast majority of the literature-reported B-cell IVLs belong to the non-GCB subtype, expressing CD10-, Bcl-2, Bcl-6, and MUM1 positivity [61], with aberrant CD5 expression but without prognostic differences [35]. Moreover, CD34 has been proven to be a useful marker for these tumors, mainly for highlighting endovascularly located lymphoma cells [59]. Epstein–Barr virus (EBV) has infrequently been linked to IVLs, with a singular instance documented by Yamada et al. that associates EBV with IVL in the female genital system [21]. However, its pathogenetic role as well as clinical and prognostic correlations are largely unknown in these types of tumors. However, there were case reports that implied that EBV infection might be an important risk factor for poor prognosis of B-Cell IVL, similarly to the cases of diffuse large B-cell lymphoma [62]. The T-cell type was reported in one study as a lesion of the vulva [24], which was somewhat anticipated as most NK/T-cell IVLs are skin- or CNS-localized [63]. One case was reported as a T-cell-rich large B-Cell lymphoma [30]. The NK/T-cell type in the cervix and ovaries was reported in an additional study; however, as the diagnosis was established post-mortem and no prior examinations indicated involvement of these organs, it was not included in the main analysis [64].

It may be noted that certain clinical variants of IVLs display hemophagocytic lymphohistiocytosis (HLH), represented by bone marrow involvement, with a clinical syndrome of fever, hepatosplenomegaly, and thrombocytopenia. HLH is a marker of a rapid, aggressive onset and progression with a known negative prognostic impact of this syndrome on other hematological malignancies [65]. However, only a portion of the noted studies performed bone marrow aspiration whose biopsy showed hemophagocytosis [30,31,34]. Furthermore, all documented cases were discharged from the hospital in stable condition, with FDG PET/CT revealing no indications of the disease [31] or achieving complete clinical/laboratory remission [30,34].

Three examples of conjoined IVL with benign growth were recorded, including one with leiomyoma [21] and two with an endometrial polyp [25,27]. A case by Uchiyama et al. [29] reported a high-grade ovarian serous carcinoma conjoined with IVL in tumor capillaries, as proven from the resected carcinoma tissue. It should be noted that in the literature, B-cell IVL is reported in association with different neoplasms, including epithelial or soft tissue benign tumors, as well as malignant carcinomas, also known as collision tumors [66,67,68,69,70] and uterine leiomyomas; however, the latter were registered during autopsy [16]. In all of the noted cases, IVL cells were usually, if not exclusively, found within tumor-laden vessels. The exact pathophysiology of the noted phenomenon is not completely understood. Certain mechanisms have been proposed, such as alterations affecting cell-cycle regulation; for example, aberrant expression of cyclin D1 has been implicated in various human neoplasms [67]. However, cyclin D1 was proven negative multiple times in our presented cases [20,21,29].

In most documented instances of R-CHOP treatment, remission was attained at subsequent evaluations, with follow-up periods variably reported between 10 and 51 months. In general, systemic immunochemotherapy remains the principal literature-reported treatment for IVL, given that gynecological lymphomas frequently exhibit chemo-treatment sensitivity [30]. Moreover, the addition of rituximab in the chemotherapy protocol R-CHOP (compared to a protocol without rituximab) was associated with improved outcomes in reported IVL cases in Western patients, with a complete remission rate of 90%, event-free survival at 3 years reaching 89%, and overall survival at 3 years reaching 89% in [71]. Surgery is frequently claimed to be utilized for symptom control or initial diagnosis, if previous procedures failed to give a definitive diagnosis [19,20,30]. It is rarely reported as a therapeutic option for IVL [25,28]. Moreover, surgery is warranted as a curative treatment option for other conjoined uterine pathologies and masses [21,31]. Due to the disease’s rarity, treatment is determined by the unique characteristics of each patient (perhaps explaining the variation in reported treatment modalities), with both surgery and chemotherapy reported with varying results, because documented patients that passed away from the illness did not receive surgery [22,26].

The exact indication for the use of prophylaxis is not clearly specified in the presented case series [22,26,30,32,33], and this may be related either to the well-known tendency for CNS involvement reported in IVL cases [40] or to the general therapeutic approach commonly applied in diffuse large B-cell lymphomas. However, it is not a universal rule for all B-cell lymphomas, as CNS prophylaxis is tailored to risk [72] and not administered universally. Therefore, when it comes to large B-cell lymphoma in general, with varying results reported in the literature, the underlying principle is that the use of CNS prophylaxis should be considered carefully on a case-by-case basis and decided by a multidisciplinary team [73]. It should be noted that the minority of patients reported to have received stem cell transplantation [19,32] achieved remission. The application of autologous stem cell transplantation in IVL treatment remains contentious. Nevertheless, it is commonly employed for high-risk patients [74], with isolated cases documenting favorable outcomes [75].

The limitations of our study include a retrospective study design and being able to include only case reports and case series, as to our knowledge, they are the only available journal-reported cases of female genital tract IVL. The evidence predominantly comprises individual case reports, with follow-up durations being varied (primarily between 10 and 51 months) or absent, and there is a lack of consistently reported treatment data as well as the absence of specified outcome indicators (such as laboratory and imaging follow-ups, patients’ physical performance, etc.). As the presented data and conclusions are based entirely on case reports and case series, this inherently carries a high risk of selection and publication bias. There was limited, complete data on the variables that we intended to study, limiting our sample size, along with articles excluded because of their post-mortem diagnosis. Moreover, most reports had different follow-up periods and did not uniformly report imaging or diagnostic procedures, commonly not disclosing the indications for treatment protocols, which increased the inter-study heterogeneity, reducing comparability across cases.

## 5. Conclusions

IVL affecting the female genital tract is a rare condition, reported only in a small number of cases worldwide. Patients had abnormal vaginal bleeding and persistent pelvic pain in addition to B symptoms associated with lymphomas. FDG PET/CT was frequently used in conjunction with aberrant laboratory results, such as increased LDH that may indicate hematological malignancy, as these cases are often without any discernible lymphadenopathy.

Abnormal FDG uptake detected on PET/CT raised suspicion for hematologic disease affecting the female genital tract, especially in the context of the elderly with B symptoms, leading to examination/biopsy of the site for determining the diagnosis. As these IVLs were predominantly B-cell-type, the R-CHOP protocol was presented as the principal treatment approach, with hysterectomy most commonly performed for diagnostic purposes or in the presence of coexisting gynecologic pathology. While remission was documented in multiple instances over the available follow-up periods, the restricted patient sample and the variability in follow-up timelines inhibit definitive conclusions on treatment outcomes. Nonetheless, the application of R-CHOP may signify a promising therapeutic approach and could potentially enhance results relative to the historically poor prognosis linked to this condition. Ultimately, prospective controlled studies are necessary, as the existing comprehension relies exclusively on variably reported cases, which introduces the risk of selection and publishing bias, hence diminishing the robustness of the data offered.

## Figures and Tables

**Table 1:** PECOS inclusion and exclusion criteria.

Parameter	Inclusion	Exclusion
Population	Living female patients of any age diagnosed with IVL of the female genital tract	Studies reporting exclusively post-mortem or autopsy findings
Exposure	IVL of the uterus, ovarium, fallopian tube, vagina and/or vulva	IVL not affecting the female genital tract
Comparison	N/A	N/A
Outcome	Clinical, laboratory, and diagnostic findings; treatment modalities and outcomes	Incomplete demographic, clinical and diagnostic evaluation
Study Design	Case reports and case series	Review articles, editorials, animal studies and conference abstracts; non-English articles

IVL—Intravascular Lymphoma; N/A—Not Applicable.

**Table 2:** Reported cases of IVL female genital tract involvement.

Reference	Country	Title	Site	No. of Cases	Phenotype
Sur et al. (2005) [20]	Canada	Intravascular Large B-Cell Lymphoma of the Uterus: A Diagnostic Challenge	Uterus	1	Large B-Cell
Yamada et al. (2005) [21]	Japan	CD5+ Epstein–Barr virus-positive intravascular large B-cell lymphoma in the uterus co-existing with huge myoma	Uterus, Ovary	1	Large B-Cell
Lannoo et al. (2007) [22]	Belgium	Intravascular large B-cell lymphoma of the uterus presenting as fever of unknown origin (FUO) and revealed by FDG-PET.	Uterus	1	Large B-Cell
Nakamichi et al. (2007) [23]	Japan	Intravascular Lymphomatosis Initially Suspected from Uterine Cytology	Uterus	1	Large B-Cell
Zizi-Sermpetzoglou et al. (2009) [24]	Greece	Intravascular T-cell lymphoma of the vulva, CD30 positive: a case report	Vulva	1	T-Cell
Yamamoto et al. (2011) [25]	Japan	Intravascular Large B-Cell Lymphoma of the Uterus: A Case with Favorable Clinical Outcome	Uterus	1	Large B-Cell
Hemmaway et al. (2012) [26]	UK	FDG-PET guided diagnosis of vaginal intravascular diffuse large B-cell lymphoma	Vagina	1	Large B-Cell
Yan Xia et al. (2014) [27]	China	Intravascular large B-cell lymphoma of the endometrium.	Uterus	1	Large B-Cell
Shigematsu et al. (2016) [28]	Japan	Intravascular Large B-cell Lymphoma of the Bilateral Ovaries and Uterus in an Asymptomatic Patient with a t(11; 22)(q23; q11) Constitutional Translocation	Uterus, ovaries	1	Large B-Cell
Uchiyama et al. (2016) [29]	Japan	Intravascular Large B-Cell Lymphoma Coexisting with an Ovarian Carcinoma	Ovary	1	Large B-Cell
Hadjadj et al. (2017) [19]	France	Uterine intravascular lymphoma as a cause of fever of unknown origin	Uterus, Ovary	5	Large B-Cell
Johannesson et al. (2017) [30]	Australia	Primary Non-Hodgkin’s Lymphomas of the Uterus and Uterine Cervix	Uterus	1	Large B-Cell
Wang et al. (2017) [31]	USA	Uterine intravascular large B-cell lymphoma presenting as abnormal uterine bleeding	Uterus	1	Large B-Cell
Alrabeh and Hao (2019) [32]	USA	“Double-expressor” intravascular large B-cell lymphoma involving the female genital tract	Uterus, FT, Ovary	1	Large B-Cell
Ong et al. (2021) [33]	Taiwan	Intravascular Large B-cell lymphoma: A case series and review of the literature	Uterus	1	Large B-Cell
Seo et al. (2025) [34]	South Korea	18F fluorodeoxyglucose PET/CT in intravascular large B-cell lymphoma involving the uterus	Uterus	1	Large B-Cell

FDG-PET—fluorodeoxyglucose positron emission tomography; USA—United States of America; UK—United Kingdom; FT—Fallopian Tube.

**Table 3:** Pelvic region FDG PET/CT abnormalities, with concordant examinations and histology reports.

Ref.	Abnormal FDG Uptake	Examination	Histology Report
[22]	Uterus	Hysteroscopy and curettage	Intravascular large B-cell lymphoma
[26]	Uterus, cervix, vagina	Vaginal mucosal biopsy	Intravascular large B-cell lymphoma
[28]	Uterus (fundus)	Cytological examination	Non-epithelial malignant cells
		Hysterectomy	Intravascular large B-cell lymphoma
[19]	Heterogeneous body uptake	Hysteroscopic endometrial biopsy	Positive
	Homogeneous body uptake	Hysteroscopic endometrial biopsy	Normal
		Hysterectomy	Intravascular lymphoma
	Diffuse uterine uptake	Punch biopsy of skin lesions	Intravascular lymphoma
	Heterogeneous body uptake	Punch biopsy of skin lesions	Intravascular lymphoma
	Diffuse uterine uptake	Hysteroscopic endometrial biopsy	Normal
		Coelioscopic iliac adenopathy biopsy	Intravascular lymphoma
[30]	Uterus	Curettage Hysterectomy	Large cell lymphoma Intravascular large B-cell lymphoma

FDG—fluorodeoxyglucose.

**Table 4:** Reported IVL immunohistochemistry.

Ref.	CD20	CD79a	CD5	CD10	BCL-2	BCL-6	MUM-1	Ki-67	Other
[20]	+	+	+	−	+	+	+	80–85%	CD23−, CD30−, CD34−, CD43−, CyclinD1−
[21]	+	+	+	−	N/A	N/A	N/A	N/A	CD45+, CD3−, CD34-Lambda+, CyclinD1−, EBV+
[23]	+	+	N/A	N/A	N/A	N/A	N/A	N/A	CD3−
[25]	+	+	N/A	N/A	N/A	N/A	N/A	N/A	CD45+
[26]	+	+	+	N/A	N/A	N/A	N/A	100%	
[27]	+	+	−	−	+	+	+	85%	CD3−, CD23−, CD34+, FoxP1+, PAX5+
[28]	+	N/A	+	−	+	+	+	High **	CyclinD1−, SOX11−
[29]	+	+	+	−	+	−	+	N/A	CyclinD1−
[19]	+	N/A	N/A	−	N/A	N/A	+	N/A	N/A
[31] *	+	+	+	−	N/A	N/A	N/A	N/A	CD3−, CD34+, PAX5+
[32]	+	N/A	+	+	+	N/A	N/A	90%	MYC+

* Immunohistochemistry reported for only one case (out of five reported) that underwent hysterectomy; ** Exact % of Ki-67 is not given.

**Table 5:** Main treatment modalities and reported outcomes.

Reference	Treatment	Outcome (Months Where Reported)
[20]	R-CHOP, surgery *	RM (14)
[21]	R-CHOP, surgery *	RM (10)
[22]	R-CHOP, CNOP-R	DOD after the second chemotherapy cycle
[23]	R-CHOP	RM
[25]	R-CHOP, surgery *	RM (51)
[26]	R-CHOP	DOD **
[27]	Not reported	RM (10)
[28]	R-CHOP, surgery *	RM (10)
[29]	Left adnexectomy, chemotherapy (not specified), radiation	DOD
[19]	All 5 cases received R-CHOP	RM in 4 cases (25, 36, 36, 36), 1 patient DOD (3)
[30]	R-CVP, R-Hyper CVAD, surgery *	RM
[31]	R-CHOP, surgery *	PET/CT scan showed no evidence of lymphoma. ***
[32]	R-EPOCH	RM
[33]	R-CHOP, surgery *	RM (13+ months)
[34]	R-CHOP, surgery *	RM

* Surgery encompasses total hysterectomy (THO) with or without bilateral salpingo-oophorectomy (BSO); ** the patient developed neutropenic sepsis and died 2 weeks after diagnosis; *** THO-BSO showed endometrial carcinoma and the patient died within 8 months after initial diagnosis; CHOP, cyclophosphamide, doxorubicin, vincristine, and prednisone; R-CHOP, rituximab plus CHOP; R-CVP, rituximab, cyclophosphamide, vincristine, and prednisone; R-EPOCH, rituximab, etoposide, prednisone, vincristine, cyclophosphamide, and doxorubicin; R-Hyper CVAD, rituximab plus hyperfractionated cyclophosphamide, vincristine, doxorubicin, and dexamethasone; CNOP-R, cyclophosphamide, mitoxantrone, vincristine, prednisone, and rituximab; RM, remission; DOD, died of the disease.

## Data Availability

No new data were created or analyzed in this study. Data sharing is not applicable to this article.

## Supplementary Materials

The following supporting information can be downloaded at: https://www.mdpi.com/article/10.3390/diseases14030109/s1, Table S1. Clinical and laboratory features of reported female genital tract IVL cases. Table S2. Treatment and Reported Outcomes of female genital tract IVL cases.

## Abbreviations

The following abbreviations are used in this manuscript:

IVL	Intravascular Lymphoma
NHL	Non-Hodgkin’s Lymphoma
CNS	Central Nervous System
WHO	World Health Organization
FDG PET/CT	Fluorodeoxyglucose Positron Emission Tomography Computed Tomography
PECOS	Population, Exposure, Comparison, Outcomes and Study Design
N/A	Not Applicable
USA	United States of America
FT	Fallopian Tube
LDH	Lactate Dehydrogenase
CRP	C-Reactive Protein
sIL-2	Soluble Interleukin-2 Receptor
CT	Computed Tomography
MRI	Magnetic Resonance Imaging
THO	Total Hysterectomy
BSO	Bilateral Salpingo-Ovariectomy
CHOP	Cyclophosphamide, Doxorubicin, Vincristine, and Prednisone
R-CHOP	Rituximab plus CHOP
R-CVP	Rituximab, Cyclophosphamide, Vincristine, and Prednisone
R-EPOCH	Rituximab, Etoposide, Prednisone, Vincristine, Cyclophosphamide, and Doxorubicin
R-Hyper CVAD	Rituximab Plus Hyperfractionated Cyclophosphamide, Vincristine, Doxorubicin, and Dexamethasone
CNOP-R	Cyclophosphamide, Mitoxantrone, Vincristine, Prednisone, and Rituximab
RM	Remission
DOD	Died of the Disease
HLH	Hemophagocytic Lymphohistiocytosis
