# Supplementary material for: Intravascular Lymphoma Associated with the Female Genital Tract—Diagnostic Considerations, Therapeutic Approaches, and Outcomes
Source: Diseases. 2026 Mar 17;14(3):109. doi: 10.3390/diseases14030109 (PMC13025139; doi:10.3390/diseases14030109)
Supplement: Supplementary file 1 [file diseases-14-00109-s001.zip › Supplementary Table S1.pdf]

Supplementary Table S1. Clinical and laboratory features of reported female genital tract IVL cases.

Ref.	Age	Clinical	Laboratory
[20]	62	Postmenopausal bleeding	LDH, serum immunoglobulins and prostatic acid phosphatase (PAP) were normal
[21]	42	general fatigue, weight loss, and abdominal fullness; genital bleeding.	Anemia, normal platelets and WBC;<br>Slighly elevated serum CRP, elevated sIL-2R, normal LDH
[22]	63	Fever, night sweats, weight loss	Anemia, normal WBC and platelet count, elevated CRP, elevated LFTs
[23]	59	Fever and general fatigue	Elevated LDH, elevated LFT, elevated sIL2-R
[24]	48	Fever, myalgia, arthralgia, weakness, altered mental status, confusion	
[25]	71	Fever and edema of both the lower legs; irregular genital bleeding	Anemia, thrombocytopenia, leukopenia;<br>elevated LDH, elevated sIL-2R
[26]	76	Anorexia and weight loss; urinary and faecal incontinence with saddle anaesthesia.	Elevated LDH and and erythrocyte sedimentation rate with elevated IgM paraprotein
[27]	66	Poor sleep and appetite with weight loss; chronic cough, malaise; vaginal bleeding without obvious cause.	Reduction in the percentage of lymphocytes (16.3%), elevation of the percentage of monocytes (22.1%). Low MCH, elevated CRP. Hemagglutinin routine test showed that PT Activity degree (PA) was reduced, activated partial thromboplastin time (APTT) and APTT ratio (APTT R) were both elevated.
[28]	72	Asymptomatic	significant increase in LDH;
[29]	61	Fever, night sweats, weight loss; neurological symtoms and deficits (motor and sensory)	Anemia, elevated LDH, borderline CRP
[19]	75	Fever	Anemia, increased CRP, hyperferritinemia
	66	Fever, confusion, gait disorder	Anemia, increased CRP and LDH, hyponatremia, hyperferritinemia, hypoalbuminemia
	59	Fever, Rash	Anemia, thrombocytopenia, increased LDH, hyponatremia, hyperferritinemia, cholestasis
	66	Fever, Oculomotor palsy, rash	Anemia, increased CRP
	67	Fever, Panhypopituitarism	Anemia, thrombocytopenia, increased CRP and LDH, hyperferritinemia
[30]	61	Weight loss and night sweats	Elevated LDH
[31]	55	Altered mental status, weakness, fatigue; weight loss;<br>Abnormal uterine bleeding	Pancytopenia, no blasts or lymphoma cells. Elevated LDH; no abnormalities in the serum electrolyte, LFT and KFT.
[32]	43	Fever, chronic pelvic pain	Anemia, elevated LDH
[33]	62	Fever, limbs edema	Anemia, elevated LDH
[34]	73	Fever	Anemia, thrombocytopenia

LDH, Lactate Dehydrogenase; CRP, C-Reactive Protein; sIL2-R. soluble Interleukin 2; LFT - liver function tests; MCH, Mean corpuscular hemoglobin; KFT, kidney function tests.
