# Supplementary material for: Intravascular Lymphoma Associated with the Female Genital Tract—Diagnostic Considerations, Therapeutic Approaches, and Outcomes
Source: Diseases. 2026 Mar 17;14(3):109. doi: 10.3390/diseases14030109 (PMC13025139; doi:10.3390/diseases14030109)
Supplement: Supplementary file 1 [file diseases-14-00109-s001.zip › Supplementary Table S2.pdf]

Table S2. Treatment and Reported Outcomes of female genital tract IVL cases.

Reference	Treatment	Outcome (months)
[20]	THO-BSO, R-CHOP	RM (14)
[21]	THO-BSO, R-CHOP, followed by high-dose etoposide plus filgrastim for peripheral blood stemcell	RM (10)
[22]	R-CHOP, CNOP-R, polychemotherapy for CNS lymphoma*	DOD after the second chemotherapy cycle
[23]	R-CHOP	RM
[25]	THO-BSO, R-CHOP	RM (51)
[26]	R-CHOP, intrathecal methotrexate	DOD**
[27]	Not reported	RM (10)
[28]	R-CHOP	RM (10)
[29]	Left adnexectomy, Chemotherapy (not precised), Radiation	DOD
[19]	All 5 cases recieved R-CHOP;<br>2 cases had autologous stem cell transplantation;<br>1 patient had DHAOx ibrutinib and oral temozolomide.	RM achieved in 4 cases (25,36,36,36), 1 patient DOD (3)
[30]	THO-BSO, treated with R-CVP with intrathecal methotrexate as initial debulking therapy followed by six cycles of R-Hyper CVAD.	RM
[31]	R-CHOP, THO-BSO	A post-therapy PET scan showed no evidence of lymphoma. ***
[32]	R-EPOCH and prophylactic intrathecal methotrexate followed by an autologous stem cell transplant.	RM
[33]	Histerectomy, R-CHOP and intrathecal therapy with methotrexate.	RM, 13+ months
[34]	THO-BSO, R-CHOP	RM

*Polychemotherapy for CNS lymphoma: BCNU, Methotrexate, ARA-C and Vincristine (BVAM); ** developed neutropenic sepsis and died 2 weeks after diagnosis; ***HO-BSO showed endometrial carcinoma, died within 8 months on initial diagnosis. THO-BSO, total hysterectomy with bilateral salpingo-oophorectomy; CHOP, cyclophosphamide, doxorubicin, vincristine, and prednisone; R-CHOP, rituximab plus CHOP; R-CVP, rituximab, cyclophosphamide, vincristine, and prednisone; R-EPOCH, rituximab, etoposide, prednisone, vincristine, cyclophosphamide, and doxorubicin; R-Hyper-CVAD, rituximab plus hyperfractionated cyclophosphamide, vincristine, doxorubicin, and dexamethasone; CNOP-R, cyclophosphamide, mitoxantrone, vincristine, prednisone, and rituximab; DHAOx, dexamethasone, high-dose cytarabine, and oxaliplatin; CNS, central nervous system; RM, remission; DOD, Died of the Disease.
